# SNVHMM: predicting single nucleotide variants from next generation sequencing

**DOI:** 10.1186/1471-2105-14-225

**Published:** 2013-07-15

**Authors:** Jiawen Bian, Chenglin Liu, Hongyan Wang, Jing Xing, Priyanka Kachroo, Xiaobo Zhou

**Affiliations:** 1 School of Mathematics and Physics, China University of Geosciences, Wuhan 430074, China; 2 Department of Radiology, The Methodist Hospital Research Institute, Weill Cornell Medical College, Houston 77030 TX, USA; 3 Department of Statistics, Hubei University of Economics, Wuhan, 430025, China; 4 Department of Diagnostic Radiology, Center for Bioinformatics & Systems Biology, Wake Forest University - School of Medicine, Winston-Salem 27103, NC, USA

## Abstract

**Background:**

The rapid development of next generation sequencing (NGS) technology provides a novel avenue for genomic exploration and research. Single nucleotide variants (SNVs) inferred from next generation sequencing are expected to reveal gene mutations in cancer. However, NGS has lower sequence coverage and poor SNVs detection capability in the regulatory regions of the genome. Post probabilistic based methods are efficient for detection of SNVs in high coverage regions or sequencing data with high depth. However, for data with low sequencing depth, the efficiency of such algorithms remains poor and needs to be improved.

**Results:**

A new tool SNVHMM basing on a discrete hidden Markov model (HMM) was developed to infer the genotype for each position on the genome. We incorporated the mapping quality of each read and the corresponding base quality on the reads into the emission probability of HMM. The context information of the whole observation as well as its confidence were completely utilized to infer the genotype for each position on the genome in study. Therefore, more probability power can be gained over the Bayes based methods, which is very useful for SNVs detection for data with low sequencing depth. Moreover, our model was verified by testing against two sets of lobular breast tumor and Myelodysplastic Syndromes (MDS) data each. Comparing against a recently published SNVs calling algorithm SNVMix2, our model improved the performance of SNVMix2 largely when the sequencing depth is low and also outperformed SNVMix2 when SNVMix2 is well trained by large datasets.

**Conclusions:**

SNVHMM can detect SNVs from NGS cancer data efficiently even if the sequence depth is very low. The training data size can be very small for SNVHMM to work. SNVHMM incorporated the base quality and mapping quality of all observed bases and reads, and also provides the option for users to choose the confidence of the observation for SNVs prediction.

## Background

In recent years, the advent of NGS technology has largely propelled the genomic research. NGS can generate millions of reads ranging from 30–350 base pairs (bp) based on the sequencing platform used. Continuous improvement in NGS technology brings the increasing of the throughput to a high extent and also lowers the cost [1]. With abundant reads aligned, many novel inferences can be made including regulatory element identification, mutation detection, gene expression estimation and detection of RNA splicing and fusion transcripts. NGS is expected to be a powerful tool for revealing genetic variations contributing to various complex diseases by providing sequence of a set of candidate genes, the whole exome or the whole genome. For example, whole genome sequencing can help in finding the frequency of tumor-specific point mutations for diseases such as multiple myeloma [2], while whole exome sequencing can be used to discover protein-coding mutation as well as small non-coding RNAs and aberrant transcriptional regulation that may contribute to diseases such as MDS [3].

The SNV calling algorithms can be divided into two categories. The first category includes threshold based commercial software packages such as Roche GSMapper and Lasergene, and the second category entails posterior probability based method including Maq [4], SOAPsnp [5], Varscan [6], Atlas-SNP2 [7] etc. For the threshold based prediction methods, a good threshold setting is difficult to obtain and relies heavily on the user experience [8].

In transcriptome based data, the number of reads representing a given transcript is highly variable across all genes making it difficult to determine a minimum depth. Moreover, the confidence for the prediction of each location is unavailable. Compared to the threshold based methods, posterior probability (Bayes) based methods achieve flexibility by considering the confidence of observation of each position on the genome. For the cancer genome sequencing data, sequencing errors, as well as the altered ploidy and tumor cellularity, are important factors affecting the accuracy of SNV calling. Although tools exist for SNVs discovery from NGS data, few are specifically suited to work with data from tumors. Recently, SNVMix [9] addressed this problem by incorporating the dependency of near-by genotypes and the posterior probability to improve the accuracy of SNVs prediction. However, the performance of SNVMix for data with low sequencing depth is not satisfactory compared to its performance with data having high sequencing depth. It has been observed that NGS provides lower sequence coverage in certain areas of genome including regulatory regions [10]. It is necessary to improve the performance of SNVs detection for tumor data with low sequencing depth. Moreover, SNVMix has achieved a relatively high sensitivity in the Bayesian framework, but the specificity is some low. The performance of specificity is needed to be improved further.

Hidden Markov model (HMM) is widely used in many fields such as speech and handwriting recognition, text classification, as well as DNA and protein classification [11]. Recently, a HMM based program VARiD [12] was developed for SNVs prediction for data from multiple sequencing platforms. VARiD is mainly focused on color space sequence and does not fully consider the mapping and base quality of the aligned reads and corresponding bases on the aligned reads in the considered model. Moreover, this method is time consuming for whole genome analysis and has not been used on RNA-Seq or whole exome sequence analysis from tumor data so far.

In this paper we developed an algorithm SNVHMM, for SNVs prediction of tumor data obtained from NGS basing on a discrete HMM. Since non-SNVs are prevalent and continuous in the genome [13], point mutations in cancer data are relevant to certain genes and are concentrated in the corresponding area [14,15], the contextual information, especially for the non-SNVs, can be considered and made full use of in addition to the information from the overall distribution of traditional Bayesian framework. So SNVHMM is expected to gain more probability power from the contextual information on the genome compared to traditional Bayesian framework, and obtain better performance for SNVs prediction. Moreover, with the contextual information added to the whole distribution information, SNVHMM is also expected to improve the statistical performance of Bayesian method for tumor data with low sequencing depth.

## Implementation

### Problem formulation and SNVHMM model specification

**Figure 1:**
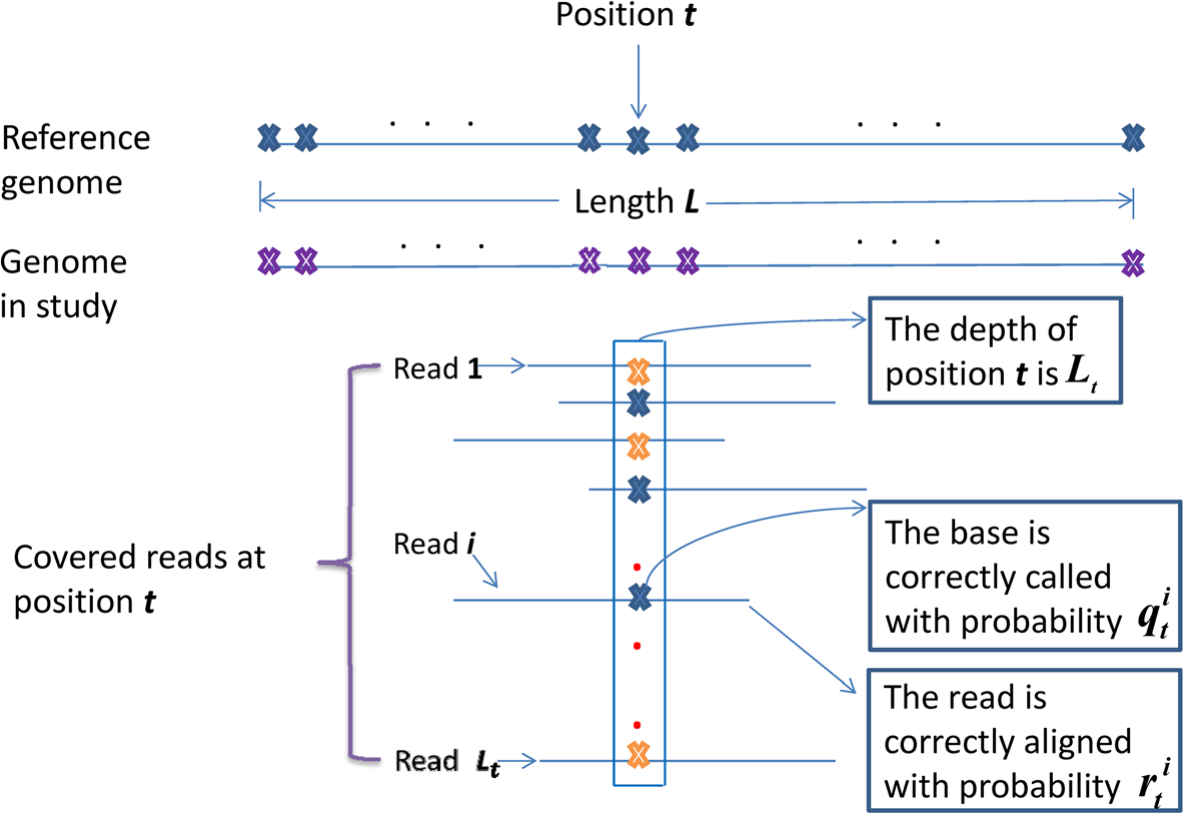
**Observation of HMM: the illustration for alignment at position *t *for the sequence in study.** The observation sets ${\left\{r_{i}^{t}\right\}}_{i=1}^{L_{t}}$ and ${\left\{q_{i}^{t}\right\}}_{i=1}^{L_{t}}$ are considered as the observation of *o*_*t*_ in HMM. For the bases on the covered reads, blue color denotes the base is the same as reference allele, yellow color denotes the base is different from reference allele while purple color denotes the base is undecided on the genome in study.

We denote the length of the considered genome as *L*. Given the aligned reads for the sequence in study, we can get the depth *L*_*t*_ of the stated position *t* on the genome. The quality of the reads covering position *t* and the quality of corresponding bases on the reads are denoted as ${\left\{r_{i}^{t}\right\}}_{i=1}^{L_{t}}$ and ${\left\{q_{i}^{t}\right\}}_{i=1}^{L_{t}}$ respectively (Figure 1). We consider three genotypes for each stated position as {aa, ab, bb}, where {aa} denotes homozygous for the reference allele, {ab} denote heterozygous and {bb} denote homozygous for the non-reference allele. Our aim is to predict the genotype for each position on the genome, given the aligned reads.

**Figure 2:**
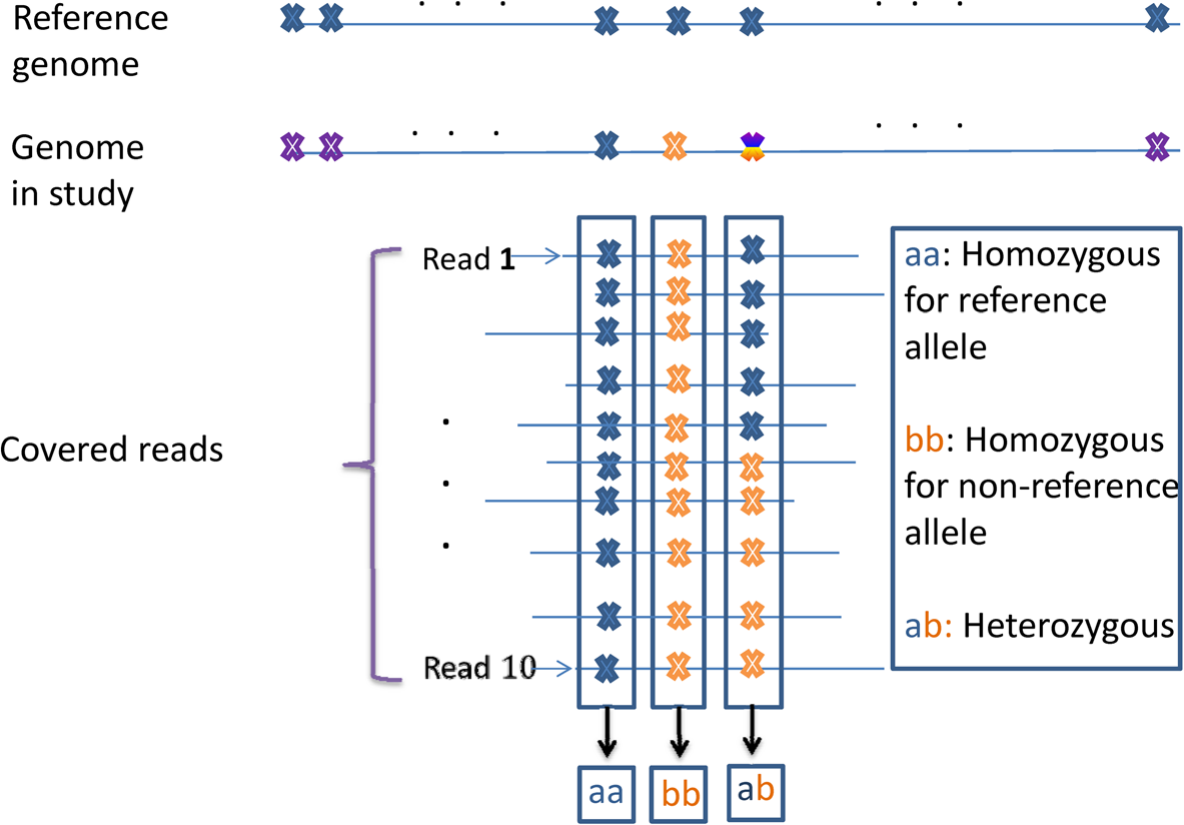
**States of HMM: the illustration for three states {aa}, {ab} and {bb} in HMM. ** The meaning of different colors are defined the same as in Figure 1.

We denote the number of the hidden states as *I*. The hidden state and observation for each position are noted as ***S*** = {*s*_*t*_}(*t* = 1, 2, ⋯, *L*) ∈ {*v*_*i*_}(*i* = 1, 2, ⋯, *I*) and ***O*** = {***o***_*t*_}(*t* = 1, 2, ⋯, *L*) respectively, where ${\left\{v_{i}\right\}}_{i=1}^{I}$ are all states considered. The underlying genotypes of the sequenced genome are taken as the hidden states, which are interpreted as follows: (1) homozygous for normal; (2) heterozygous; (3) homozygous for mutation (Figure 2). These states are important in detecting single nucleotide polymorphism or point mutation for normal sample as well as cancer sample. The last two states are taken as SNV in our study. For simplicity, we note state {aa} as state 1, state {ab} as state 2 and state {bb} as state 3 in the following initial state distribution and state transition matrix.

• Initial state distribution:

$$\pi =\left\{\pi_{1},\pi_{2},\pi_{3}\right\},\;\pi_{i}=P\left(s_{1}=v_{i}|t=1\right)$$

• State transition matrix:

$$A=\left(\begin{array}{ccc} a_{11} & a_{12} & a_{13}\\ a_{21} & a_{22} & a_{23}\\ a_{31} & a_{32} & a_{33} \end{array}\right)≜\left(A_{1}^{T},A_{2}^{T},A_{3}^{T}\right),$$

$$a_{\mathrm{ij}}=P\left(s_{t+1}=v_{j}|s_{t}=v_{i}\right)$$

• Emission probability distribution:

$$B=\left\{b_{s_{t}}\left(o_{t}\right)\right\}$$

The observation to be considered for each position includes the coverage, the mapping quality of the covering reads and the base quality on the covering reads corresponding to each stated position. The observation for each position *t* is taken as $o_{t}={\left\{q_{i}^{t},r_{i}^{t}\right\}}_{i=1}^{L_{t}}$. The emission probability $b_{v_{i}}\left(o_{t}\right)$ is calculated as a conditional probability, given the hidden state:

(1) $$b_{s_{t}}\left(o_{t}\right)=P\left({\left\{q_{i}^{t},r_{i}^{t}\right\}}_{i=1}^{L_{t}}|s_{t}=v_{i}\right)=f\left({\left\{q_{i}^{t},r_{i}^{t}\right\}}_{i=1}^{L_{t}}\right)$$

To make full use of the mapping quality and base quality for each position on the genome, we compute $b_{s_{t}}\left(o_{t}\right)$ using the whole probability formula by considering if the covered reads are correctly aligned and if the corresponding bases on these reads are correctly called. We use a formula motivated by (5) in [9] by introducing a generalized Binomial distribution in addition to the conditional computation of the base calling probability and aligning probability.

(2) $$b_{v_{i}}\left(o_{t}\right)=\left(\begin{array}{l} L_{t}\\ P_{t} \end{array}\right)\prod_{j=1}^{L_{t}}\left\{0.25\left(1-r_{j}^{t}\right)+0.5r_{j}^{t}\left[q_{j}^{t}u_{i}+\left(1-q_{j}^{t}\right)\left(1-u_{i}\right)\right]\right\}$$

where ${\left\{u_{i}\right\}}_{i=1}^{I}$ is the Binomial distribution parameter for each position on the genome and *P*_*t*_ is the number of reads having the same base with reference allele at position *t*. The detailed derivation of (2) is given at the supplementary file. In this study, we only considered two types of nucleotides covering the stated position, which have the largest and second largest number at the stated position. In the case of rare third alleles, these reads are assumed to be errors. In this study, *u*_*i*_ denotes the probability of occurrence for the allele having the largest number at the stated position.

### Prior distribution of HMM

We take the initial distribution of *π* as Dirichlet distribution with hyper-parameter ***δ*** = (*δ*_1_, *δ*_2_, *δ*_3_), ***u*** = (*u*_1_, *u*_2_, *u*_3_) is taken conjugately according to a Beta distribution with hyper-parameter ***α*** = (*α*_1_, *α*_2_, *α*_3_) and ***β*** = (*β*_1_, *β*_2_, *β*_3_) as follows:

(3) $$P\left(\pi |\delta \right)=\text{Dirichlet}\left(\pi |\delta \right)$$

(4) $$P\left(u_{k}|\alpha_{k},\beta_{k}\right)=\text{Beta}\left(u_{k}|\alpha_{k},\beta_{k}\right)$$

where we take ***δ*** = (1000, 100, 100) by assuming that most positions will be homozygous for the reference allele. We also set ***α*** = (1000, 500, 1) and ***β*** = (1, 500, 1000) by assuming the probability of state {aa} occurring at the stated position is much larger than that it not occurring, vice versa for state {bb}. We also assume the probability of state {ab} occurring at the stated position is the same as that it not occurring. For the initial distribution of state transition matrix, we take the initial distribution of *A*_*i*_ as follows:

(5) $$P\left(A_{i}|\gamma_{i}\right)=\text{Dirichlet}\left(A_{i}|\gamma_{i}\right)$$

where we take ***γ***_***1***_ = (1000, 100, 100), ***γ***_***2***_ = (100, 1000, 100) and ***γ***_***3***_ = (100, 100, 1000)*.* Since the sum of elements in *A*_*i*_ should be equal to probability 1, a normalization for ${\left\{A_{i}\right\}}_{i=1}^{I}$ is performed after each iteration of SNVHMM.

### Estimation of HMM parameters

For simplicity, we denote the model parameters of HMM as ***λ*** ≜ (***π***, ***u***, ***A***) and learn the unknown HMM by using EM algorithm and computing the maximum likelihood estimation when the observed data are incomplete [16]. The aim is to find the model parameter *λ* maximizing the observation probability i.e. *L*(***o***, ***λ***) ≜ P(***o***|***λ***) or log P(***o***|***λ***),where the later one is usually used when the length of the observation is large. We use a special case of EM algorithm, Baum-Welch algorithm [11], to learn the unknown parameters. For the training of HMM, we use the following auxiliary function $Q\left(\lambda ,\bar{\lambda }\right)$ as the objective function for the optimization of the HMM parameters.

(6) $$Q\left(\lambda ,\bar{\lambda }\right)=\Sigma_{s\in S}\text{log}P\left(O,S|\bar{\lambda }\right)P\left(S|O,\lambda \right)$$

It is proved that maximizing the following auxiliary function can lead to the increase of the likelihood P(***O***|***λ***), i.e. $\frac{\text{max}}{\bar{\lambda }}Q\left(\lambda ,\bar{\lambda }\right)\to P\left(O|\bar{\lambda }\right)>P\left(O|\lambda \right)$[11]. Given model parameter set *λ*, P(***O***, ***S***|***λ***) can be calculated as:

(7) $$P\left(O,S|\lambda \right)=\pi_{s_{0}}\prod_{t=1}^{L}a_{s_{t-1}s_{t}}b_{s_{t}}\left(o_{t}\right)$$

Replacing term P(***O***, ***S***|***λ***) in (6) with (7), (6) can be rewritten as:

(8) $$\begin{array}{ll} Q\left(\lambda ,\bar{\lambda }\right)= & \sum_{s\in S}\text{log}{\bar{\pi }}_{s_{0}}P\left(O,S|\lambda \right)+\sum_{s\in S}\left(\sum_{t=1}^{L}\text{log}{\bar{a}}_{s_{t-1}s_{t}}\right)P(O,S|\lambda )\\ & +\sum_{s\in S}\left(\sum_{t=1}^{L}\text{log}{\bar{b}}_{s_{t}}\left(o_{t}\right)\right)P\left(O,S|\lambda \right) \end{array}$$

The update of model parameters *π*_*i*_ and *a*_*ij*_ with constraints $\sum_{i=1}^{N}\pi_{i}=1$ and $\sum_{j=1}^{N}a_{\mathrm{ij}}=1$ can be obtained by maximizing the first and second term of (8) with respect to *π*_*i*_ and *a*_*ij*_ respectively as follows:

(9) $${\bar{\pi }}_{i}=\frac{P\left(O,s_{0}=i|\lambda \right)}{P\left(O|\lambda \right)}$$

(10) $${\bar{a}}_{\mathrm{ij}}=\frac{\sum_{t=1}^{T}P\left(O,s_{t-1}=i,s_{t}=j|\lambda \right)}{\sum_{t=1}^{T}P\left(O,s_{t-1}=i|\lambda \right)}$$

The update for ${\left\{u_{i}\right\}}_{i=1}^{I}$ can be obtained by maximizing the third term with respect to ${\left\{u_{i}\right\}}_{i=1}^{I}$, however, the close-form expression is not available due to the complicated structure of the observation term $f\left({\left\{q_{i}^{t},r_{i}^{t}\right\}}_{i=1}^{L_{t}}\right)$. We use a Newton iteration with respect to the first and second derivation of the third term as follows:

(11) $$u_{i}^{\text{new}}=u_{i}^{\text{old}}-\frac{\sum_{t=1}^{T}\frac{\partial f}{\partial u_{i}}*\frac{P\left(O,s_{t}=i|\lambda \right)}{f}}{\frac{\partial f}{\partial u_{i}}\left\{\sum_{t=1}^{T}\frac{\partial f}{\partial u_{i}}*\frac{P\left(O,s_{t}=i|\lambda \right)}{f}\right\}}$$

The forward and backward algorithm [11] is used to update *π*_*i*_ and *a*_*ij*_. In the implementation of Baum-Welch, the update of *u*_*i*_ is used for the update of the emission probability. Finally, we use Viterbi algorithm [11] to infer the hidden states of the sequence in study.

## Results

### Dataset

Two types of tumor data are used to verify the effectiveness of our model. The first type is the lobular breast tumor data with two different sequencing depths, which includes 497 positions generated using the Illumina GA II platform and was validated by Sanger. These positions were sequenced using Sanger capillary-based technology and were predicted to be non-synonymous protein-coding. 305 of these positions were confirmed as SNV and are taken as positive (TP), while 192 were not confirmed and are taken as true negative (TN). We take these positions as ground truth for the computation of TP, false positive (FP), TN, false negative (FN). These data can be obtained from the supplementary dataset 2A and 2C [9] along with their corresponding ground truth for SNVs in supplementary dataset 2B and 2D. The depths of supplementary dataset 2A and 2C are 10X and 40X respectively. Moreover, we use these datasets to compare between SNVHMM and SNVMix2, which is more efficient than SNVMix1 [9]. For better training of SNVMix2, we also use the supplementary dataset 3A and 3C [9].

The second dataset that came from MDS tumor data comprises of 7 MDS samples including 5 samples from RNA-Seq having depth <20 and 2 samples from whole exome sequencing having depth >150. These data are all from our lab and 4 mutated MDS genes were validated by PCR, along with other 23 common mutated MDS genes [14,15] were also checked by SNVHMM for point mutation detection on these genes.

### Statistical metrics

We take states of ‘ab’ and ‘bb’ as SNV for each location on the genome. Accuracy, sensitivity, specificity and F-score are proposed to evaluate the performance of SNVHMM and SNVMix2, which are defined as follows:

$$\text{Accuracy}=\frac{\mathrm{TP}+\mathrm{TN}}{\mathrm{TP}+\mathrm{FP}+\mathrm{TN}+\mathrm{FN}},\;\text{Sensitivity}=\frac{\mathrm{TP}}{\mathrm{TP}+\mathrm{FN}}$$

Specificity=TNTN+FP,F=2Precision×RecallPrecision+Recall

where $\text{Precision}=\frac{\mathrm{TP}}{\mathrm{TP}+\mathrm{FP}}$ and $\text{Recall}=\frac{\mathrm{TP}}{\mathrm{TP}+\mathrm{FN}}$, are the proportion of true SNVs being predicted among the total predicted positives and the total true positives respectively.

### Statistical performance

**Figure 3:**
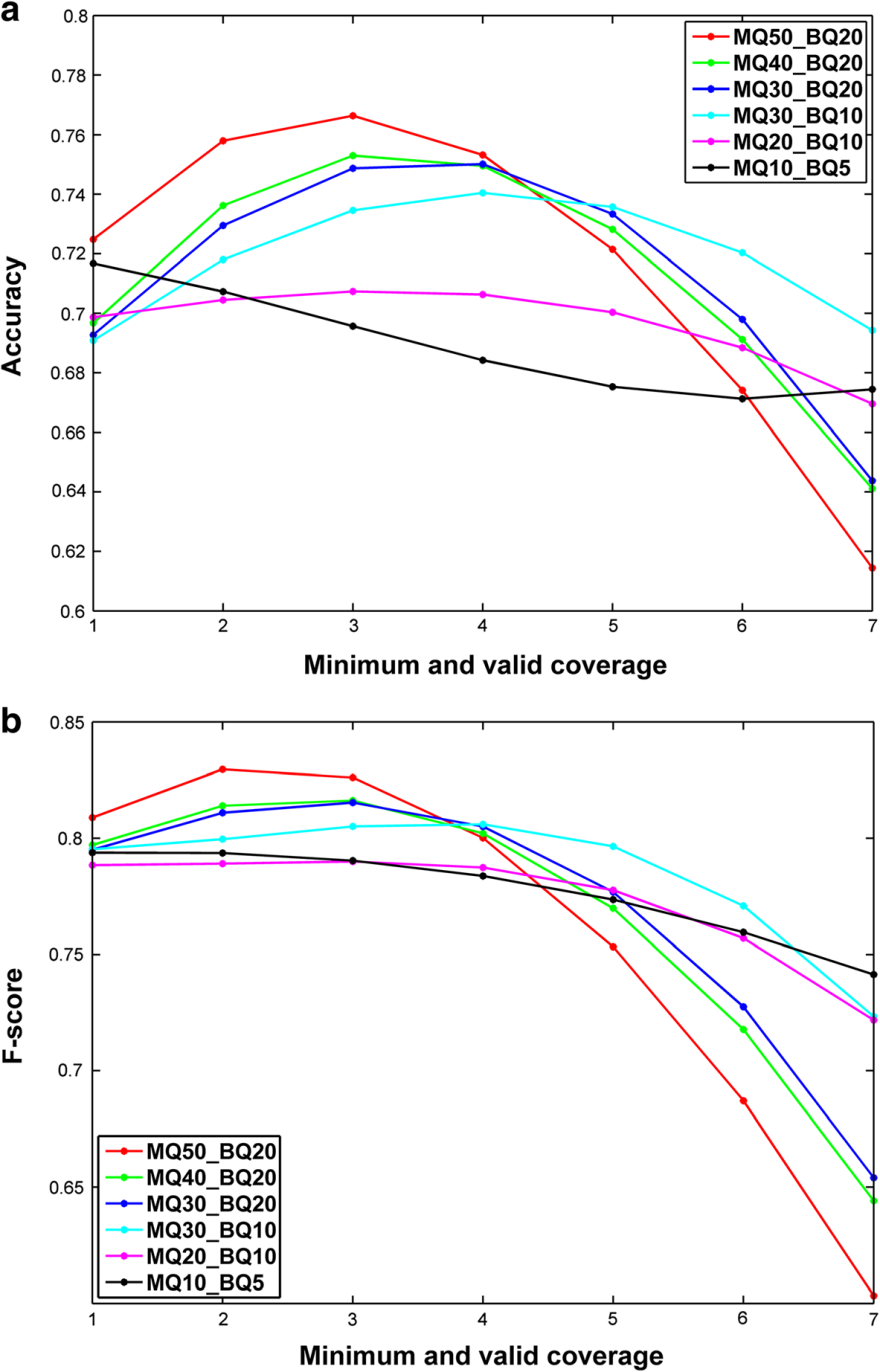
Plot of the (a) accuracy and (b) F-score with the change of minimum and valid value of coverage for 10X lobular breast cancer data.

**Figure 4:**
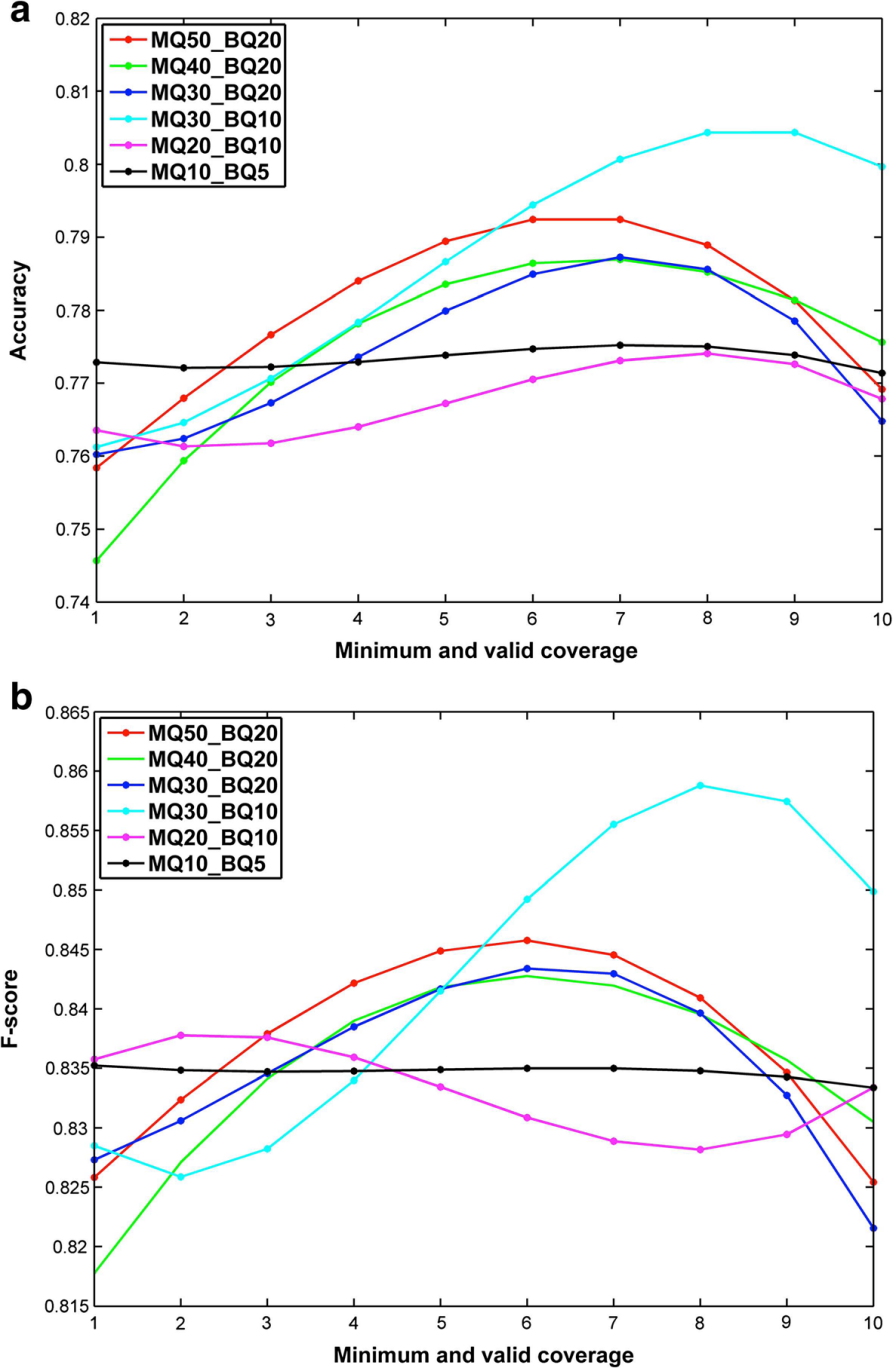
Plot of the (a) accuracy and (b) F-score with the change of minimum valid value of coverage for 40X lobular breast cancer data.

We compare the sensitivity, specificity, precision and F-score between SNVHMM and SNVMix2 on one lobular breast tumor data with sequencing depth 10X and 40X respectively. To get better classification results for SNVMix2, we used the supplementary datasets 3A and 3C [9] for training (SNVMix2_TO) which includes 14649 positions on different chromosomes. To test the gain of ability for SNVHMM in utilizing contextual information in addition to the information of whole distribution in SNVMix2, we train SNVHMM only by the test dataset itself, which is much smaller than the datasets 3A and 3C. For comparison purpose, we also tested SNVMix2 by training it on datasets 2A and 2C for 10X and 40X lobular breast tumor data respectively (SNVMix2_TI). The initial parameter for SNVHMM is set up as described in Section Prior distribution of HMM. Although we have specified how to encode base and mapping uncertainty into the emission probability in SNVHMM, obviating the need for taking thresholds for these quantities. However, different threshold setting of mapping quality (MQ) and base quality (BQ) as well as minimum and valid coverage (MVC) for each location enable us to achieve better performance. Here, MVC is the number of the least reads used to support the prediction of SNV. The results of SNVHMM and SNVMix2 for 10X and 40X lobular breast tumor data under different MQ and BQ condition are reported in Tables 1 and 2 respectively. The results are an average estimator basing on 20 independent runs for SNVHMM and SNVMix2 under different threshold settings. For each MQ and BQ condition, we choose the MVC achieving the best precision result for SNVHMM. The precision and F-score of SNVHMM with respect to different MVC for 10X and 40X lobular breast tumor data are plotted in Figure 3a, b and Figure 4a, b respectively. It is observed from Table 1 that SNVHMM performs significantly better than SNVMix2_TI and gains about 10% for precision and 3% for F-score in average. SNVHMM also outperforms SNVMix2_TO for nearly all the MQ and BQ conditions except MQ30_BQ10, although the gain for F-score is not obvious. It is also observed that SNVHMM decreases the false positive rate and increases the true negative rate compared with SNVMix2_TI and SNVMix2_TO, which leads to the improvement of specificity with a gain of 10% in average over SNVMix2_TO and 51% in average over SNVMix2_TI while maintaining a relatively high sensitivity. The same trend can also be observed in Table 2. SNVHMM outperforms both SNVMix2_TO and SNVMix2_TI for nearly all threshold conditions. Comparing SNVHMM with SNVMix2_TI, SNVHMM gains about 3.5% for precision and 14% for specificity. Comparing SNVHMM with SNVMix2_TO, SNVHMM gains about 2.1% for precision and 8.7% for specificity while maintaining a relatively high sensitivity. It is also noted that the performance of SNVHMM attains its peak and also achieves the best gain over SNVMix2_TI and SNVMix2_TO at MQ30&BQ10 condition.

**Table 1:** Comparison of statistical performance of SNVHMM with SNVMix2 with different mapping quality (MQ) and base quality (BQ) threshold for 10X data

**Model**	**MQ**	**BQ**	**TP**	**FP**	**TN**	**FN**	**Sensitivity(%)**	**Specificity(%)**	**Accuracy(%)**	**F-score**
SNVHMM	50	20	247	59	133	58	80.98	69.27	76.46(MVC = 4)	0.8085
	40	20	254	66	126	51	83.28	65.63	76.46(MVC = 4)	0.8128
	30	20	256	70	112	49	83.93	65.54	76.05(MVC = 4)	0.8114
	30	10	236	63	129	69	77.38	67.19	73.44(MVC = 5)	0.7815
	20	10	273	111	81	32	89.51	42.19	71.23(MVC = 3)	0.7925
	10	5	273	110	82	32	89.51	42.71	71.43(MVC = 2)	0.7936
SNVMix2_TI	50	20	303	160	32	2	99.34	16.67	67.40	0.7891
	40	20	305	174	18	0	100	9.38	64.99	0.7781
	30	20	305	174	18	0	100	9.38	64.99	0.7781
	30	10	305	173	19	0	100	9.89	65.19	0.7791
	20	10	305	191	1	0	100	0.52	61.56	0.7615
	10	5	305	192	0	0	100	0	61.37	0.7606
SNVMix2_TO	50	20	245	75	117	60	80.32	60.94	72.84	0.7840
	40	20	261	88	104	44	85.57	54.17	73.44	0.7982
	30	20	266	90	102	39	87.21	53.13	74.04	0.8048
	30	10	274	92	100	31	89.83	52.08	75.25	0.8167
	20	10	283	125	67	22	92.78	34.90	70.42	0.7938
	10	5	290	134	58	15	95.08	30.21	70.02	0.7956

**Table 2:** Comparison of statistical performance of SNVHMM with SNVMix2 with different mapping quality (MQ) and base quality (BQ) threshold for 40X data

**Model**	**MQ**	**BQ**	**TP**	**FP**	**TN**	**FN**	**Sensitivity(%)**	**Specificity(%)**	**Accuracy(%)**	**F-score**
SNVHMM	50	20	281	77	115	24	92.13	59.89	79.68(MVC = 7)	0.8477
	40	20	283	83	109	22	92.78	56.77	78.87(MVC = 6)	0.8435
	30	20	273	77	115	32	89.51	59.89	78.07(MVC = 9)	0.8336
	30	10	289	79	113	16	94.75	58.85	80.88(MVC = 9)	0.8588
	20	10	279	86	106	26	91.47	55.21	77.46(MVC = 8)	0.8328
	10	5	281	87	105	24	92.13	54.69	77.67(MVC = 7)	0.8351
SNVMix2_TI	50	20	291	109	83	14	95.40	43.23	75.25	0.8255
	40	20	294	113	79	11	96.39	41.14	75.05	0.8258
	30	20	294	115	77	11	96.39	40.10	74.65	0.8235
	30	10	295	113	79	10	96.72	41.15	75.25	0.8275
	20	10	294	117	75	11	96.39	39.06	74.25	0.8212
	10	5	295	118	74	10	96.72	38.54	74.25	0.8217
SNVMix2_TO	50	20	283	86	106	22	92.79	55.21	78.27	0.8398
	40	20	287	93	99	18	94.10	51.56	77.67	0.8380
	30	20	287	96	96	18	94.10	50.00	77.06	0.8343
	30	10	284	105	87	21	93.11	45.31	74.65	0.8184
	20	10	291	105	87	14	95.40	45.31	76.06	0.8302
	10	5	291	104	88	14	95.41	45.83	76.26	0.8314

**Figure 5:**
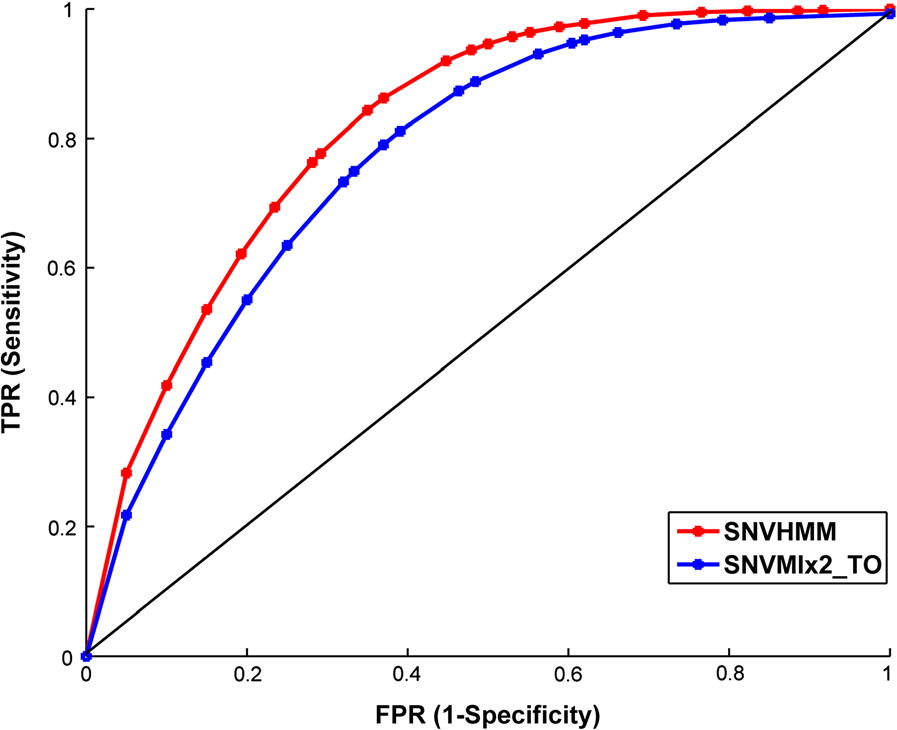
ROC curve of SNVHMM and SNVMix2_TO for lobular breast cancer data.

Comparing Table 1 with Table 2, we can observe the overall improvement for performance of SNVHMM, SNVMix2_TI and SNVMix2_TO. It is not surprising for more information can be used for SNVs prediction with the increase of sequencing depth. It is also noticed that the performance gap between SNVMix2_TI and SNVMix2_TO narrowed with the increase of sequencing depth while the gain of accuracy for SNVHMM over SNVMix2_TI is obvious. From Figures 3 and 4, it is observed that the best MVC value for precision and F-score is between 2–5 for 10X data and between 6–9 for 40X data respectively at different threshold conditions. So a moderate MVC ranging from 15% to 25% in proportion to the sequencing depth is needed for efficient prediction under different threshold settings. It is not surprising as a small MVC value can result in estimating some locations with low confidence and a large MVC value can cause some locations with high confidence being excluded from prediction. The performance of SNVHMM with respect to different MQ and BQ threshold and MVC condition is presented in Additional file 1: Table S1 and Table S2 for 10X and 40X data respectively. We also compute the p-value of accuracy and F-score for SNVHMM against SNVMix2_TI and SNVMix2_TO for different MQ and BQ threshold settings. For SNVMix2_TI and SNVHMM with sequencing depth 10X, the p-values for accuracy and F-score are less than 1.8e-23 and 3.2e-4 respectively (ANOVA, t-test), while the p-values for accuracy and F-score are less than 1.5e-12 and 9.9e-3 respectively (ANOVA, t-test) for SNVMix2_TI and SNVHMM with sequencing depth 40X. For SNVMix2_TO and SNVHMM with sequencing depth 10X, the p-values for accuracy and F-score are less than 1.3e-23 and 8.8e-12 respectively (ANOVA, t-test), while the p-values for accuracy and F-score are less than 1.9e-7 and 4.1e-5 respectively (ANOVA, t-test) for SNVMix2_TI and SNVHMM with sequencing depth 40X. So the gain of SNVHMM over SNVMix2_TI is statistical significant for both 10X and 40X data while SNVHMM is also significantly better than SNVMix2_TO, although the absolute gain of SNVHMM over SNVMix2_TO is limited. The ROC of SNVHMM and SNVMix2_TO for 20 independent runs over different MQ and BQ threshold are plotted in Figure 5, which show an obvious improvement for SNVHMM against SNVMix2_TO. Since the performance of SNVMix2_TO is better than SNVMix2_TI, the plot of ROC for SNVMix2_TI is not presented here.

### Implementation and robust analysis

The proposed algorithm is implemented in C and supports both Maq [4] and SAMtools [17] pileup format. Running SNVHMM on the lobular breast cancer data with sequencing depth 40X takes 1 ~ 2 seconds and it needs ~20 seconds for the lobular breast cancer data including 14649 locations with sequencing depth 40X on 64 bit Linux Ubuntu 3.0.0. SNVHMM is robust under different MQ and BQ threshold settings. The standard deviations of accuracy and F-score are between 0.001 and 0.003 respectively for both 10X and 40X lobular breast cancer data.

The software is available online at https://sites.google.com/site/snvhmm4/. The initial setting and trained parameters are also available for the lobular breast cancer data and MDS data.

### Performance of SNVHMM on MDS sample

To test the effectiveness of SNVHMM on large-size tumor data, we use SNVHMM on two groups of MDS samples to explore some common mutated genes for MDS. The two groups of data include 5 RNA-Seq samples and 2 whole exome samples. We take MQ = 50, BQ = 20 and MVC = 4 for RNA_Seq data and take MQ = 50, BQ = 20 and MVC = 7 for the whole exome data. We use ANNOVAR [18] for the annotation of point mutations. The number of point mutations detected by SNVHMM is reported in Table 3 and the corresponding annotated genes are reported in Additional file 1: Table S3. 18 common MDS mutated genes from [14] and 5 common MDS mutated genes from [15] are checked. Moreover, 4 MDS genes are validated by our lab. The validated information is presented in Additional file 1: Table S4. It can be seen that majority of the 27 genes in Additional file 1: Table S3 were detected in either RNA-Seq data or whole exome data. Some MDS mutated genes are detected in only few samples or not detected such as IDH1, IDH2 and PTPN11, which are proved to be rare in MDS [19]. Finally, 4 new mutated MDS genes: MLL3, IQGAP2, DIDO1 and EIF4G2 are all detected by SNVHMM to be non-synonymous, which coincides with our validated result.

**Table 3:** Number of point mutations found in 5 MDS RNA-Seq data and 2 whole exome data for SNVHMM

**Type**	**RNA-Seq**					**Whole exome**	
Sample	RS_1	RS_2	RS_3	RS_4	RS_5	WE_1	WE_2
Number^1^	10645(91.6%)	33354(93.3%)	13881(91.5%)	4777(94.2%)	6951(92.6%)	58803(94.8%)	61344(93.7%)

^1^The proportion that both SNVHMM and SNVMix2 predicted is reported in the parenthesis.

## Discussion

We introduced a new algorithm SNVHMM for SNVs prediction of tumor data from next generation sequencing, which generally yield data with low sequencing depth due to sequencing errors, as well as the altered ploidy and tumor cellularity. SNVHMM was conceived to circumvent the shortcomings of existing algorithms that cannot efficiently predict SNVs for data with low sequencing coverage. In this algorithm, we considered three genotypes concerned as the hidden states of HMM, and incorporated the confidence of the observation into the emission probability in HMM. The performance of SNVHMM was compared with a recently published method SNVMix2. Compared to SNVMix2, SNVHMM considered the relation of state from near-by locations in addition to their distribution. Moreover, SNVHMM predicted the hidden states by maximizing the posterior probability in condition of the whole observation while SNVMix2 predicted the genotype basing on maximizing the posterior probability in condition of the observation from single location. So SNVHMM gained more probability power for prediction from the same dataset. It was shown by experiment from the lobular breast cancer data sequenced with lower depth that SNVHMM improved the performance of SNVMix2 by only using much smaller size of training data. It was also observed that SNVHMM even exceeded the performance of SNVMix2 trained by much larger datasets. If looking into the performance of SNVHMM and SNVMix2_TI for lobular cancer data, we found that SNVHMM corrected 42% ~ 75% and 26% ~ 33% false positives to true negatives for sequencing depth 10X and 40X respectively, with more than 85% of them to have coverage less than 20. For SNVHMM and SNVMix2_TO, SNVHMM also corrected 10% ~ 17% and 2% ~ 8% false positives to true negatives for sequencing depth 10X and 40X respectively, with more than 80% of them to have coverage less than 20. So SNVHMM improved the performance of SNVMix2 for low-coverage sequencing data or at the low depth area of genome by improving the true negative rate largely. This verified the effectiveness of SNVHMM in utilizing the contextual information of non-SNVs by improving the specificity largely while remaining a relatively high sensitivity.

For experiments on MDS samples, SNVHMM could detect the point mutations efficiently. More than 95% of the point mutations detected by both SNVHMM and SNVMix2 are obvious mutation as most of the covered reads have the same non-reference base. From the common mutated 27 genes list of MDS, most of the mutated genes can be found in majority of the samples by SNVHMM. We also examined the region of the genes not detected to find no non-reference bases covered or insufficient non-reference bases covered with low quality.

For the training of the HMM parameters, we give the parameters before and after training for the lobular breast cancer data and MDS data in Table 4. The threshold of mapping quality and base quality are taken as 50 and 20 respectively and the initial setting of the parameters are taken from the distribution as defined in the “Prior distribution of HMM” Section. For the lobular breast cancer data, we both considered the 10X and 40X conditions. It is observed that majority of the parameters changed significantly while only a few did not change much. The trained *π* differs largely from the initial value for the lobular breast cancer data. It is not surprising as *π* indicates the proportion of three kinds of bases on the genome, and it is observed from the ground truth of the lobular breast cancer data that majority of the bases have the state “ab”. It is also observed that majority parameters of the trained *u* and *A* changed significantly. The initial setting of *u* and *A* seems to be some close to the true parameters of these data. For the MDS data, the trained *π* and *A* changed largely compared with the initial setting while *μ* also changed but not as much as *π* and *A*. We check the raw data to find that the SNVs are sparse and state “aa” dominates the whole distribution, so it is reasonable that the first value in *π* increased largely while the second and the third value in *π* decreased. It is also not surprising that all the states have a large transition probability to state “aa”. All the trained parameters for different thresholds of lobular breast cancer data and MDS data are provided at https://sites.google.com/site/snvhmm4/.

**Table 4:** Comparison of the parameters of SNVHMM before and after training on lobular breast cancer (LBC) data and MDS RNA_seq data for threshold of mapping quality 50 and base quality 20

**Parameter**		**LBC_10X**	**LBC_40X**	**MDS_RNA_Seq**
*π*	initial	(0.904233 0.499051 0.090499)	(0.904233 0.499051 0.090499)	(0.904233 0.499051 0.090499)
	trained	(0.001199 0.984717 0.014086)	(0.000001 0.999831 0.000170)	(0.988258 0.011743 0.000001)
*u*	initial	(0.999023 0.508543 0.000123)	(0.999023 0.508543 0.000123)	(0.999023 0.508543 0.000123)
	trained	(0.904833 0.466663 0.151255)	(0.897743 0.509801 0.165214)	(0.904233 0.544121 0.090499)
*A*	initial	$$\left(\begin{array}{ccc} 0.848400 & 0.072800 & 0.078800\\ 0.087570 & 0.838210 & 0.074300\\ 0.085100 & 0.075600 & 0.839300 \end{array}\right)$$	$$\left(\begin{array}{ccc} 0.848400 & 0.072800 & 0.078800\\ 0.087570 & 0.838210 & 0.074300\\ 0.085100 & 0.075600 & 0.839300 \end{array}\right)$$	$$\left(\begin{array}{ccc} 0.848400 & 0.072800 & 0.078800\\ 0.087570 & 0.838210 & 0.074300\\ 0.085100 & 0.075600 & 0.839300 \end{array}\right)$$
	trained	$$\left(\begin{array}{ccc} 0.567581\; & 0.325414 & 0.107006\\ 0.061140\; & 0.864239 & 0.074624\\ 0.044694\; & 0.257290 & 0.698019 \end{array}\right)$$	$$\left(\begin{array}{ccc} 0.412955\; & 0.476467 & 0.110580\\ 0.159858\; & 0.723625 & 0.116519\\ 0.112374\; & 0.323298 & 0.564329 \end{array}\right)$$	$$\left(\begin{array}{ccc} 0.999064\; & 0.000271 & 0.000377\\ 0.986557\; & 0.013444 & 0.000001\\ 1.000000\; & 0.000001 & 0.000001 \end{array}\right)$$

## Conclusions

We have proposed a new SNVs prediction tool SNVHMM for cancer data from NGS. SNVHMM can gain more probability power from the transition probability in additional to the posterior probability computation for the genotype distribution of whole observation. So SNVHMM is very efficient when the depth of NGS data is very low. Since NGS has lower sequence coverage and poor SNV detection capability in the regulatory regions of the genome, it is very helpful for SNV prediction for the low-depth area on the genome. SNVHMM outperformed an existing SNV prediction tool SNVMix by reducing its false positives and increasing its true negative. Moreover, SNVHMM needs much less data for training while obtaining a better performance than SNVMix. Finally, two types of MDS data with different coverage are tested, which shows the effectiveness of SNVHMM.

## Availability and requirements

** Project name:** SNVHMM: predicting single nucleo tide variants from next generation sequencing

**Project home page:** https://sites.google.com/site/snvhmm4/

**Operating system:** 64-bit Linux

**Programming language:** C

**Other requirements:** Linux Ubuntu 3.0.0 or higher

**License:** GNU GPL

**Any restrictions to use by non-academics:** license  needed.

## Competing interests

The authors declare that they have no competing interests.

## Authors’ contributions

JB and XZ performed the design of the study. JB, CL, JX and HW participated in the implementation of the algorithm. CL and JB prepared and analyzed the breast and MDS data. JB drafted the manuscript. PK revised the manuscript. All authors read and approved the final manuscript.

## Supplementary Material

Additional file 1: Table S1 Statistical performance of SNVHMM for different minimum and valid coverage (d), as well as for different MQ and BQ value when the sequencing depth of lobular breast cancer data is 10X. **Table S2:** Statistical performance of SNVHMM for different minimum and valid coverage (d), as well as for different MQ and BQ value when the sequencing depth of lobular breast cancer data is 40X. **Table S3:** 23 reported mutated genes in Bejar,R. et al. (2011) and Thol,F. et al. (2012) are checked by SNVHMM. 4 new genes that are found in 5 MDS RNA-Seq sample and 2 MDS whole exome samples are found by SNVHMM and validated by our lab. **Table S4:** description of 4 MDS-related mutated genes found by SNVHMM and validated by our lab in 5 RNA-Seq and 2 whole exome samples. Click here for file
