# Supplementary material for: SNVHMM: predicting single nucleotide variants from next generation sequencing
Source: BMC Bioinformatics. 2013 Jul 15;14:225. doi: 10.1186/1471-2105-14-225 (PMC3718670; doi:10.1186/1471-2105-14-225)
Supplement: Additional file 1: Table S1 — Statistical performance of SNVHMM for different minimum and valid coverage (d), as well as for different MQ and BQ value when the sequencing depth of lobular breast cancer data is 10X. Table S2: Statistical performance of SNVHMM for different minimum and valid coverage (d), as well as for different MQ and BQ value when the sequencing depth of lobular breast cancer data is 40X. Table S3: 23 reported mutated genes in Bejar,R. et al. (2011) and Thol,F. et al. (2012) are checked by SNVHMM. 4 new genes that are found in 5 MDS RNA-Seq sample and 2 MDS whole exome samples are found by SNVHMM and validated by our lab. Table S4: description of 4 MDS-related mutated genes found by SNVHMM and validated by our lab in 5 RNA-Seq and 2 whole exome samples. [file 1471-2105-14-225-S1.doc]

# Supplementary file for paper “SNVHMM: predicting single nucleotide variants from next generation sequencing ”

### Jiawen Bian^1,2^, Chenglin Liu^2^, Hongyan Wang^2^, Jing Xing^2^, Priyanka Kachroo^2^ and Xiaobo Zhou^3§^

^1^School of Mathematics and Physics, China University of Geosciences, Wuhan, China, 430074

^2^Department of Radiology, The Methodist Hospital Research Institute, Weill Cornell Medical College, Houston, TX 77030, USA

^3^ Department of Diagnostic Radiology, Center for Bioinformatics & Systems Biology, Wake Forest University - School of Medicine, Winston-Salem, NC 27103, USA

^§^Corresponding author

**Derivation of Equation (2)**

For derivation purpose, we introduce some notations as follows:

$a_{j}^{t}$, indicates whether read $j$ at position $t$matches the reference allele,

$b_{j}^{t}$, indicates whether read $j$aligns to its stated position

So according to the observation of $\{q_{i}^{t},r_{i}^{t}\}_{i=1}^{L_{t}}$, we have :

$$P(b_{j}^{t}=1)=q_{j}^{t},P(b_{j}^{t}=0)=1-q_{j}^{t};$$

$$P(b_{j}^{t}=0,a_{j}^{t}=0)=0.5,P(b_{j}^{t}=0,a_{j}^{t}=1)=0.5;$$

$$P(b_{j}^{t}=1,a_{j}^{t}=1)=q_{j}^{t},P(b_{j}^{t}=1,a_{j}^{t}=0)=1-q_{j}^{t};$$

From the observation structure, a generalized binomial distribution can be used to calculate the emission probability of HMM by using the total probability formula as follows:

$$\begin{matrix} P(o_{t}|v_{i})=\left(\begin{matrix} L_{t} \\ P_{t} \end{matrix}\right)\prod_{j=1}^{L_{t}} \sum_{a_{j}^{t}} \sum_{b_{j}^{t}} P(o_{t}a_{j}^{t}b_{j}^{t}|v_{i}) \\ =\left(\begin{matrix} L_{t} \\ P_{t} \end{matrix}\right)\prod_{j=1}^{L_{t}} \sum_{a_{j}^{t}} \sum_{b_{j}^{t}} P(o_{t}|a_{j}^{t}b_{j}^{t}v_{i})P(a_{j}^{t}|b_{j}^{t}v_{i})P(v_{i}|b_{j}^{t})P(b_{j}^{t}) \\ =\left(\begin{matrix} L_{t} \\ P_{t} \end{matrix}\right)\prod_{j=1}^{L_{t}} \left\{\begin{matrix} P(o_{t}|\{a_{j}^{t}=1\}\{b_{j}^{t}=1\}v_{i})P(\{a_{j}^{t}=1\}|\{b_{j}^{t}=1\}v_{i})P(v_{i}|\{b_{j}^{t}=1\})P(\{b_{j}^{t}=1\})+ \\ P(o_{t}|\{a_{j}^{t}=0\}\{b_{j}^{t}=1\}v_{i})P(\{a_{j}^{t}=0\}|\{b_{j}^{t}=1\}v_{i})P(v_{i}|\{b_{j}^{t}=1\})P(\{b_{j}^{t}=1\})+ \\ P(o_{t}|\{a_{j}^{t}=1\}\{b_{j}^{t}=0\}v_{i})P(\{a_{j}^{t}=1\}|\{b_{j}^{t}=0\}v_{i})P(v_{i}|\{b_{j}^{t}=0\})P(\{b_{j}^{t}=0\})+ \\ P(o_{t}|\{a_{j}^{t}=0\}\{b_{j}^{t}=0\}v_{i})P(\{a_{j}^{t}=0\}|\{b_{j}^{t}=0\}v_{i})P(v_{i}|\{b_{j}^{t}=0\})P(\{b_{j}^{t}=0\}) \end{matrix}\right\} \\ =\left(\begin{matrix} L_{t} \\ P_{t} \end{matrix}\right)\prod_{j=1}^{L_{t}} \left\{0.25(1-r_{j}^{t})+0.5r_{j}^{t}[q_{j}^{t}u_{i}+(1-q_{j}^{t})(1-u_{i})]\right\} \end{matrix}$$

**Supplementary Table 1**: Statistical performance of SNVHMM for different minimum and valid coverage (d), as well as for different MQ and BQ value when the sequencing depth of lobular breast cancer data is 10X

| MQ | BQ | d | TP | FP | TN | FN | Sensitivity | Specificity | Accuracy | F-measure |
| --- | --- | --- | --- | --- | --- | --- | --- | --- | --- | --- |
| 50 | 20 | 1 | 291 | 122 | 70 | 14 | 95.41 | 36.46 | 72.64 | 0.8106 |
|  |  | 2 | 289 | 104 | 88 | 16 | 94.75 | 45.83 | 75.86 | 0.8281 |
|  |  | 3 | 272 | 87 | 105 | 33 | 89.18 | 54.69 | 75.86 | 0.8193 |
|  |  | 4 | 247 | 59 | 133 | 58 | 80.98 | 69.27 | 76.46 | 0.8085 |
|  |  | 5 | 217 | 50 | 142 | 88 | 71.15 | 73.96 | 72.23 | 0.7587 |
|  |  | 6 | 173 | 34 | 158 | 132 | 56.72 | 82.23 | 66.60 | 0.6758 |
|  |  | 7 | 147 | 32 | 160 | 158 | 48.19 | 83.33 | 61.77 | 0.6074 |
| 40 | 20 | 1 | 296 | 142 | 50 | 9 | 97.05 | 26.04 | 69.62 | 0.7968 |
|  |  | 2 | 286 | 110 | 82 | 19 | 93.77 | 42.71 | 74.04 | 0.8160 |
|  |  | 3 | 275 | 98 | 94 | 30 | 90.16 | 48.96 | 74.25 | 0.8112 |
|  |  | 4 | 251 | 65 | 127 | 54 | 82.30 | 66.15 | 76.06 | 0.8084 |
|  |  | 5 | 224 | 56 | 136 | 81 | 73.44 | 70.83 | 72.43 | 0.7658 |
|  |  | 6 | 197 | 46 | 146 | 108 | 64.59 | 76.04 | 69.01 | 0.7190 |
|  |  | 7 | 161 | 34 | 158 | 144 | 52.78 | 82.29 | 64.19 | 0.6440 |
| 30 | 20 | 1 | 296 | 144 | 48 | 9 | 97.05 | 25 | 69.22 | 0.7946 |
|  |  | 2 | 289 | 116 | 76 | 16 | 94.75 | 39.58 | 73.44 | 0.8141 |
|  |  | 3 | 276 | 102 | 90 | 29 | 90.49 | 46.88 | 73.64 | 0.8082 |
|  |  | 4 | 256 | 70 | 122 | 49 | 83.93 | 63.54 | 76.06 | 0.8114 |
|  |  | 5 | 229 | 56 | 136 | 76 | 75.08 | 70.83 | 73.44 | 0.7763 |
|  |  | 6 | 202 | 50 | 142 | 103 | 66.23 | 73.96 | 69.22 | 0.7253 |
|  |  | 7 | 167 | 38 | 154 | 138 | 54.75 | 80.21 | 64.59 | 0.6549 |
| 30 | 10 | 1 | 295 | 147 | 45 | 10 | 96.72 | 23.44 | 68.41 | 0.7898 |
|  |  | 2 | 288 | 115 | 77 | 17 | 94.42 | 40.10 | 73.44 | 0.8136 |
|  |  | 3 | 267 | 96 | 96 | 38 | 87.54 | 50 | 73.04 | 0.7994 |
|  |  | 4 | 268 | 98 | 94 | 37 | 87.87 | 48.96 | 72.84 | 0.7988 |
|  |  | 5 | 256 | 83 | 109 | 49 | 83.93 | 56.77 | 73.44 | 0.7950 |
|  |  | 6 | 236 | 63 | 129 | 69 | 77.38 | 67.19 | 73.44 | 0.7815 |
|  |  | 7 | 198 | 48 | 144 | 107 | 64.92 | 75 | 68.81 | 0.7187 |
| 20 | 10 | 1 | 278 | 123 | 69 | 27 | 91.15 | 35.94 | 69.82 | 0.7875 |
|  |  | 2 | 277 | 119 | 73 | 28 | 90.82 | 38.02 | 70.42 | 0.7903 |
|  |  | 3 | 273 | 111 | 81 | 32 | 89.51 | 42.19 | 71.23 | 0.7925 |
|  |  | 4 | 270 | 114 | 78 | 35 | 88.52 | 40.63 | 70.02 | 0.7837 |
|  |  | 5 | 257 | 101 | 91 | 48 | 84.26 | 47.40 | 70.02 | 0.7753 |
|  |  | 6 | 245 | 93 | 99 | 60 | 80.32 | 51.56 | 69.22 | 0.7621 |
|  |  | 7 | 212 | 72 | 120 | 93 | 69.51 | 62.50 | 66.80 | 0.7199 |
| 10 | 5 | 1 | 273 | 110 | 82 | 32 | 89.51 | 42.71 | 71.43 | 0.7936 |
|  |  | 2 | 273 | 110 | 82 | 32 | 89.51 | 42.71 | 71.43 | 0.7936 |
|  |  | 3 | 292 | 140 | 52 | 13 | 95.74 | 27.08 | 69.22 | 0.7924 |
|  |  | 4 | 284 | 139 | 53 | 21 | 93.11 | 27.60 | 67.81 | 0.7802 |
|  |  | 5 | 273 | 126 | 66 | 32 | 89.51 | 34.38 | 68.21 | 0.7756 |
|  |  | 6 | 259 | 118 | 74 | 46 | 84.92 | 38.54 | 67.00 | 0.7595 |
|  |  | 7 | 232 | 89 | 103 | 73 | 76.07 | 53.65 | 67.40 | 0.7412 |

**Supplementary Table 2**: Statistical performance of SNVHMM for different minimum and valid coverage (d), as well as for different MQ and BQ value when the sequencing depth of lobular breast cancer data is 40X

| MQ | BQ | d | TP | FP | TN | FN | Sensitivity | Specificity | Accuracy | F-measure |
| --- | --- | --- | --- | --- | --- | --- | --- | --- | --- | --- |
| 50 | 20 | 1 | 287 | 103 | 89 | 18 | 94.10 | 46.35 | 75.65 | 0.8259 |
|  |  | 2 | 284 | 92 | 100 | 21 | 93.11 | 52.08 | 77.26 | 0.8341 |
|  |  | 3 | 284 | 91 | 101 | 21 | 93.11 | 52.60 | 77.46 | 0.8353 |
|  |  | 4 | 283 | 85 | 107 | 22 | 92.79 | 55.73 | 78.47 | 0.8410 |
|  |  | 5 | 293 | 96 | 96 | 12 | 96.07 | 50 | 78.27 | 0.8444 |
|  |  | 6 | 283 | 79 | 113 | 22 | 92.79 | 58.85 | 79.68 | 0.8486 |
|  |  | 7 | 281 | 77 | 115 | 24 | 92.13 | 59.90 | 79.68 | 0.8477 |
|  |  | 8 | 277 | 78 | 114 | 28 | 90.82 | 59.38 | 78.67 | 0.8394 |
|  |  | 9 | 268 | 73 | 119 | 37 | 87.87 | 61.98 | 77.87 | 0.8297 |
|  |  | 10 | 275 | 84 | 108 | 30 | 90.16 | 56.25 | 77.06 | 0.8283 |
| 40 | 20 | 1 | 285 | 106 | 86 | 20 | 96.44 | 44.79 | 74.65 | 0.8190 |
|  |  | 2 | 285 | 99 | 93 | 20 | 93.44 | 48.44 | 76.06 | 0.8273 |
|  |  | 3 | 285 | 97 | 95 | 20 | 93.44 | 49.48 | 76.46 | 0.8297 |
|  |  | 4 | 285 | 89 | 103 | 20 | 93.44 | 53.65 | 78.07 | 0.8395 |
|  |  | 5 | 292 | 95 | 97 | 13 | 95.74 | 50.52 | 78.27 | 0.8439 |
|  |  | 6 | 283 | 83 | 109 | 22 | 92.79 | 56.77 | 78.87 | 0.8435 |
|  |  | 7 | 285 | 84 | 108 | 20 | 93.44 | 56.25 | 79.07 | 0.8457 |
|  |  | 8 | 278 | 81 | 111 | 27 | 91.14 | 57.82 | 78.26 | 0.8373 |
|  |  | 9 | 272 | 78 | 114 | 33 | 89.18 | 59.38 | 77.67 | 0.8305 |
|  |  | 10 | 276 | 81 | 111 | 29 | 90.49 | 57.81 | 77.87 | 0.8338 |
| 30 | 20 | 1 | 288 | 103 | 89 | 17 | 94.42 | 46.35 | 75.86 | 0.8276 |
|  |  | 2 | 286 | 97 | 95 | 19 | 93.77 | 49.48 | 76.66 | 0.8314 |
|  |  | 3 | 286 | 97 | 95 | 19 | 93.77 | 49.48 | 76.66 | 0.8314 |
|  |  | 4 | 277 | 87 | 105 | 28 | 90.82 | 54.69 | 76.86 | 0.8407 |
|  |  | 5 | 287 | 91 | 101 | 18 | 94.10 | 52.60 | 78.07 | 0.8404 |
|  |  | 6 | 287 | 87 | 105 | 18 | 94.10 | 54.69 | 78.87 | 0.8454 |
|  |  | 7 | 285 | 85 | 107 | 20 | 93.44 | 55.73 | 78.87 | 0.8444 |
|  |  | 8 | 278 | 82 | 110 | 27 | 91.15 | 57.29 | 78.07 | 0.8361 |
|  |  | 9 | 273 | 77 | 115 | 32 | 89.51 | 59.90 | 78.07 | 0.8336 |
|  |  | 10 | 270 | 82 | 110 | 35 | 88.52 | 57.29 | 76.46 | 0.8219 |
| 30 | 10 | 1 | 295 | 111 | 81 | 10 | 96.72 | 42.19 | 75.65 | 0.8298 |
|  |  | 2 | 271 | 79 | 113 | 34 | 88.85 | 58.85 | 77.26 | 0.8275 |
|  |  | 3 | 271 | 79 | 113 | 34 | 88.85 | 58.85 | 77.26 | 0.8275 |
|  |  | 4 | 276 | 83 | 109 | 29 | 90.49 | 56.77 | 77.46 | 0.8313 |
|  |  | 5 | 283 | 87 | 105 | 22 | 92.79 | 54.69 | 78.07 | 0.8385 |
|  |  | 6 | 294 | 89 | 103 | 11 | 96.39 | 53.65 | 79.88 | 0.8547 |
|  |  | 7 | 293 | 88 | 104 | 12 | 96.07 | 54.17 | 79.88 | 0.8542 |
|  |  | 8 | 291 | 83 | 109 | 14 | 95.41 | 56.77 | 80.48 | 0.8571 |
|  |  | 9 | 289 | 79 | 113 | 16 | 94.75 | 58.85 | 80.88 | 0.8588 |
|  |  | 10 | 285 | 81 | 111 | 20 | 93.44 | 57.81 | 79.68 | 0.8495 |
| 20 | 10 | 1 | 284 | 97 | 95 | 21 | 93.11 | 49.48 | 76.26 | 0.8280 |
|  |  | 2 | 284 | 97 | 95 | 21 | 93.11 | 49.48 | 76.26 | 0.8480 |
|  |  | 3 | 284 | 97 | 95 | 21 | 93.11 | 49.48 | 76.26 | 0.8480 |
|  |  | 4 | 276 | 89 | 103 | 29 | 90.49 | 53.65 | 76.26 | 0.8239 |
|  |  | 5 | 281 | 91 | 101 | 24 | 92.13 | 52.60 | 76.86 | 0.8301 |
|  |  | 6 | 275 | 85 | 107 | 30 | 90.16 | 55.73 | 76.86 | 0.8271 |
|  |  | 7 | 277 | 85 | 107 | 28 | 90.82 | 55.73 | 77.26 | 0.8306 |
|  |  | 8 | 279 | 86 | 106 | 26 | 91.48 | 55.21 | 77.46 | 0.8328 |
|  |  | 9 | 281 | 88 | 104 | 24 | 92.13 | 54.17 | 77.46 | 0.8338 |
|  |  | 10 | 281 | 92 | 100 | 24 | 92.13 | 52.08 | 76.66 | 0.8289 |
| 10 | 5 | 1 | 286 | 94 | 98 | 19 | 93.77 | 51.04 | 77.26 | 0.8350 |
|  |  | 2 | 286 | 94 | 98 | 19 | 93.77 | 51.04 | 77.26 | 0.8350 |
|  |  | 3 | 286 | 94 | 98 | 19 | 93.77 | 51.04 | 77.26 | 0.8350 |
|  |  | 4 | 286 | 94 | 98 | 19 | 93.77 | 51.04 | 77.26 | 0.8350 |
|  |  | 5 | 286 | 94 | 98 | 19 | 93.77 | 51.04 | 77.26 | 0.8350 |
|  |  | 6 | 280 | 87 | 105 | 25 | 91.80 | 54.69 | 77.46 | 0.8333 |
|  |  | 7 | 281 | 87 | 105 | 24 | 92.13 | 54.69 | 77.67 | 0.8351 |
|  |  | 8 | 285 | 91 | 101 | 20 | 93.44 | 52.60 | 77.67 | 0.8370 |
|  |  | 9 | 284 | 93 | 99 | 21 | 93.11 | 51.56 | 77.06 | 0.8328 |
|  |  | 10 | 283 | 91 | 101 | 22 | 92.79 | 52.60 | 77.26 | 0.8336 |

**Supplementary** **Table3**: 23 reported mutated genes in Bejar,R. et al. (2011) and Thol,F. et al. (2012) are checked by SNVHMM. 4 new gen$\{q_{i}^{t},r_{i}^{t}\}_{i=1}^{L_{t}}$es that are found in 5 MDS RNA-Seq sample and 2 MDS whole exome samples are found by SNVHMM and validated by our lab.

| Data | | RNA-Seq | | Whole Exome | |
| --- | --- | --- | --- | --- | --- |
|  |  | Number | type | Number | type |
| 18 mutated genes in Bejar,R. et al. (2011) | TET2 | 5(5) | Intron | 2(2) | Intron |
|  | ASXL1 | 4(5) | Intron | 2(2) | non-synonymous |
|  | EZH2 | 3(5) | Intron | 2(2) | Intron |
|  | TP53 | 0(5) |  | 2(2) | non-synonymous |
|  | RUNX1 | 4(5) | splicing factor | 2(2) | UTR3 |
|  | IDH1 | 1(5) | non-synonymous | 0(2) |  |
|  | IDH2 | 0(5) |  | 2(2) | Intron |
|  | NRAS | 3(5) | Intron | 2(2) | synonymous |
|  | KRAS | 1(5) | Intron | 2(2) | synonymous |
|  | CBL | 3(5) | synonymous | 2(2) | Intron |
|  | ETV6 | 5(5) | synonymous | 0(2) |  |
|  | JAK2 | 1(5) | Intron | 2(2) | Intron |
|  | NPM1 | 4(5) | Intron | 0(2) |  |
|  | GNAS | 3(5) | non-synonymous | 1(2) | non-synonymous |
|  | PTPN11 | 4(5) | Intron | 0(2) |  |
|  | PTEN | 5(5) | Intron | 2(2) | Intron |
|  | BRAF | 3(5) | Intron | 0(2) |  |
|  | CDKN2A | 0(5) |  | 2(2) | UTR3,UTR5 |
| 5 mutated genes in Thol,F. et al. (2012) | U2AF1 | 1(5) | Intron | 2(2) | Intron |
|  | DNMT3A | 1(5) | Intron | 2(2) | Intron |
|  | SRSF2 | 0(5) |  | 2(2) | synonymous |
|  | SF3B1 | 3(5) | synonymous | 2(2) | synonymous |
|  | ZRSR2 | 3(5) | synonymous | 2(2) | Intron |
| 4 validated genes by our lab | MLL3 | 3(5) | non-synonymous | 2(2) | non-synonymous |
|  | IQGAP2 | 4(5) | non-synonymous | 2(2) | non-synonymous |
|  | DIDO1 | 2(5) | non-synonymous | 2(2) | non-synonymous |
|  | EIF4G2 | 5(5) | synonymous | 2(2) | synonymous |

**Supplementary Table4**: description of 4 MDS-related mutated genes found by SNVHMM and validated by our lab in 5 RNA-Seq and 2 whole exome samples.

| Gene | Mutated code/  amino acid | novel  mutation | Function | Predict of  Functional change |
| --- | --- | --- | --- | --- |
| | IQGAP2 | | --- | | 3438G/T (Q1146H) | yes | Regulate cell morphology and motility | Damaging; likely cancer |
| | MLL3 | | --- | | 10432C/G (Q3478E) | yes | Leukemogenesis and developmental disease | Damaging; not determined |
| | DIDO1 | | --- | | 1229C/T (S410L) | yes | Involved in apoptosis | Damaging; not determined |
| | EIF4G2 | | --- | | 1618T/C (S540P) | yes | Repressor of translation | Damaging; not determined |

**Reference**

Bejar,R. et al. (2011) Clinical effect of point mutations in Myelodysplastic Syndromes. N. Engl. J. Med., **364**, 2496–2506.

Thol,F. et al. (2012) Frequency and prognostic impact of mutations in SRSF2, U2AF1, and ZRSR2 in patients with myelodysplastic syndromes. Blood, **119**: 3578–3584.
